# Efficacy and cost-effectiveness of an unguided, internet-based self-help intervention for social anxiety disorder in university students: protocol of a randomized controlled trial

**DOI:** 10.1186/s12888-019-2125-4

**Published:** 2019-06-25

**Authors:** Fanny Kählke, Thomas Berger, Ava Schulz, Harald Baumeister, Matthias Berking, Pim Cuijpers, Ronny Bruffaerts, Randy P. Auerbach, Ronald C. Kessler, David Daniel Ebert

**Affiliations:** 1 Friedrich-Alexander-University Erlangen-Nürnberg (FAU), Nägelsbachstrasse 25a, 91052 Erlangen, Germany; 2 Department of Clinical Psychology and Psychotherapy, University of Bern, Bern, Switzerland; 3 Department of Experimental Psychopathology and Psychotherapy, University of Zürich, Psychiatric University Hospital, Zürich, Switzerland; 4 Department of Clinical Psychology and Psychotherapy, University of Ulm, Ulm, Germany; 5 Department of Clinical, Neuro- and Developmental Psychology, Vrije University Amsterdam, Amsterdam, The Netherlands; 6 Research Group Psychiatry, Department of Neurosciences, KU Leuven University, Leuven, Belgium; 7 Department of Psychiatry, College of Physicians and Surgeons, Columbia University, New York, NY USA; 8 Division of Clinical Developmental Neuroscience, Sackler Institute, New York, NY USA; 9 Department for Health Care Policy, Harvard Medical School, Boston, MA USA

**Keywords:** Social anxiety disorder, Social phobia, Randomized controlled trial, Internet-based treatment, Self-help, Unguided self-help, University students, Economic evaluation

## Abstract

**Background:**

Social anxiety disorder (SAD) is highly prevalent among university students, but the majority of affected students remain untreated. Internet- and mobile-based self-help interventions (IMIs) may be a promising strategy to address this unmet need. This study aims to investigate the efficacy and cost-effectiveness of an unguided internet-based treatment for SAD among university students. The intervention is optimized for the treatment of university students and includes one module targeting fear of positive evaluations that is a neglected aspect of SAD treatment.

**Methods:**

The study is a two arm randomized controlled trial in which 200 university students with a primary diagnosis of SAD will be assigned randomly to either a wait-list control group (WLC) or the intervention group (IG). The intervention consists of 9 sessions of an internet-based cognitive-behavioral treatment, which also includes a module on fear of positive evaluation (FPE). Guidance is delivered only on the basis of standardized automatic messages, consisting of positive reinforcements for session completion, reminders, and motivational messages in response to non-adherence. All participants will additionally have full access to treatment as usual. Diagnostic status will be assessed through Structured Clinical Interviews for DSM Disorders (SCID). Assessments will be completed at baseline, 10 weeks and 6-month follow-up. The primary outcome will be SAD symptoms at post-treatment, assessed via the Social Phobia Scale (SPS) and the Social Interaction Anxiety Scale (SIAS). Secondary outcomes will include diagnostic status, depression, quality of life and fear of positive evaluation. Cost-effectiveness and cost-utility analyses will be evaluated from a societal and health provider perspective.

**Discussion:**

Results of this study will contribute to growing evidence for the efficacy and cost-effectiveness of unguided IMIs for the treatment of SAD in university students. Consequently, this trial may provide valuable information for policy makers and clinicians regarding the allocation of limited treatment resources to such interventions.

**Trial registration:**

DRKS00011424 (German Clinical Trials Register (DRKS)) Registered 14/12/2016.

## Background

Anxiety disorders have the highest prevalence compared to other mental health disorders, showing an estimated lifetime prevalence of 10–22% in European Countries [1]. Social anxiety disorder (SAD) is ranked as the third most common anxiety disorder in Germany [2], and the prevalence estimates of SAD in university students range from 3.4% (12-month) in the United States [3] to 16.1% (point-prevalence) in Sweden [4].

SAD among university students has been associated with a number of adverse effects, including low quality of life [5] and problems with identity formation [6], increased consumption of alcohol [7] and high levels of suicidal ideation [8]. Additionally, SAD-related emotional distress causes dysfunctional avoidance strategies, which are associated with underachievement and may lead to premature drop out from university [4]. Therefore, the economic burden extends beyond the direct costs of treatment to indirect costs (e.g. low productivity, increased number of sick days, lower qualification level [9, 10]) and intangible costs (e.g. reduced quality of life, social impairment). Thus, treatment of SAD is of particular interest to the public healthcare system and health services in and outside of university [11, 12], especially as SAD may become a chronic condition when left untreated [13].

Effective treatment options exist [14, 15], but are only used by a small proportion of those in need [16, 17]. Reasons for low treatment rates include not only a limited availability of trained clinicians but also other barriers to help-seeking such as fear of stigmatization. Fear of negative evaluation, the expectation that others might judge one’s behavior or physical symptoms as embarrassing or humiliating, [18, 19] is the key feature of SAD. Hence, the nature of SAD is one major reason which prevents university students from seeking professional advice [9, 20].

Internet- and mobile-based interventions are a promising strategy to reach underserved SAD populations. In contrast to traditional face-to-face therapy IMIs are immediately accessible, lack stigmatization, are more flexible, anonymous, and initiated with minimal (or no) human contact [21–23]. In addition, although the initial costs of developing an IMI can be quite high, the low marginal costs of providing IMIs to additional user are assumed to lead to lower overall expenditures [24]. Moreover, IMIs are likely to reduce health care delivery costs compared to face-to-face treatment, as IMIs involve minimal or no contact with mental health care specialists and also reduce travel costs.

A large number of studies have shown that IMIs can be effective in the treatment of common mental disorders [22]. The most recent systematic review on IMIs for SAD showed a mean standardized effect size of g = 0.84 [0.72–0.97] compared to untreated control groups and g = 0.38 [0.13–0.62] compared to active control conditions [25]. We are aware of only one small study (*n* = 38) that evaluated a psychological internet supported intervention for SAD in university students [26]. This study assessed an internet-based self-help intervention with minimal email contact to a psychotherapist with in vivo group exposure compared to no in vivo group exposure. The intervention resulted in large pre-post within-group effect-sizes for both groups. The generalizability of these results are, however, limited due to methodological shortcomings (e.g. small sample size). In addition, the study did evaluate neither the cost-effectiveness of the intervention nor the effects of unguided self-help.

One of the major cost-drivers and potential barriers for large-scale treatment dissemination is the provided level of therapeutic guidance in IMIs. In a recent meta-analysis guided IMIs yielded a mean average effect of g = 0.87 [0.72–1.02] compared to passive control conditions such as WLC (*n* = 11) [25]. The standardized effect size of unguided IMIs was g = 0.78 [0.50–1.05] compared to passive controls (*n* = 8). The effect sizes (0.28–1.47) varied widely between unguided IMIs [27–34], which makes it difficult to anticipate the expected effect size for future studies. We suspect that the variance in effect sizes between studies could be explained by methodological differences such as small (*n* = 20–40) [27, 29–32] to moderate (*n* = 56–62) [28, 34] sample sizes, different lengths of follow-ups (e.g. only three studies evaluated long-term effects [27–29]) and high dropout rates. Therefore, additional research is needed to determine the efficacy of unguided internet-based interventions in the treatment of SAD, particularly among university students.

Although it is often assumed that IMIs tend to be cost-effective, there is limited empirical evidence showing the impact on health economic outcomes [35–37]. To the best of our knowledge, only three studies investigated the cost-effectiveness of IMIs for SAD; all of these studies evaluated a therapist guided IMI [38–40]. To date, no study has investigated the cost-effectiveness of an internet-based intervention in university students, and no study has evaluated the health economic effects of an unguided internet-based intervention for SAD.

Although the efficacy of Cognitive Behavioral Therapy (CBT) in the treatment of SAD is well-documented, there is still room for improvement. Recent findings suggest not only fear of negative evaluations to be a central feature of SAD, but also prove a strong link between SAD and the fear of positive evaluation (FPE) [41, 42]. According to Weeks and Howell’s (2012) bivalent fear of evaluation model of social phobia, fear of evaluation in general is the core component of SAD, including the fear of negative (FNE) and positive evaluation [43]. The function of FPE in SAD also has been discussed in the context of evolutionary models of social anxiety [44]. Empirical evidence shows that FPE and FNE are related but distinct factors contributing to SAD, with FPE explaining a unique and independent proportion of variance in the fear of social interactions [45]. Even though established treatments for SAD do not address FPE directly, there is evidence that CBT can reduce FPE, albeit with smaller effect sizes compared to FNE [42]. And, importantly, neglecting FPE in SAD treatments may impede treatment progress (e.g., when clients still feel anxious after successful exposures that received positive feedback) [46]. Specifically, people who endorse FPE do not feel proud when making progress or achieving goals and, paradoxically, often experience discomfort [41]. In that sense, FPE often results in socially anxious people avoiding social situations in which they are the focal point (e.g., group work, presentations at university) which prevents their exposure to positive social feedback and safeguards their social status within the group [42, 47]. Although research has shown that FPE is responsive to cognitive-behavioral therapy [48], no intervention has systematically addressed this as a treatment component of SAD.

The aim of this study will be to evaluate whether an unguided internet-based intervention for SAD is effective in reducing social anxiety symptoms and other secondary outcomes such as depression, fear of positive evaluation, interpersonal problems and quality of life when compared to a WLC in university students. Additionally, cost-effectiveness analyses will be conducted from societal and health provider perspective in order to examine whether this internet-based intervention for SAD represents good value for money.

This study is part of the recently launched Caring Universities – the World Health Organization (WHO) World Mental Health International College Student (WMH-ICS) initiative (https://www.hcp.med.harvard.edu/wmh/college_student_survey.php) [49, 50]. It is an international initiative which aims to obtain accurate cross-national data on the prevalence, and correlates of mental disorders among university students throughout the world, assess unmet needs for treatment, develop practical methods to improve mental health intervention utilization, and evaluate effective strategies for the prevention and treatment of mental health disorders in university students.

## Methods

### Study design

This study is a randomized-controlled trial in which the assumed superiority of an internet-based intervention for SAD is evaluated compared to a WLC. The intervention group will receive the internet-based self-help treatment for social anxiety and the control group will obtain access to this intervention after 6 months. Both conditions have full access to university and community treatment as usual.

### Participants

**Fig. 1:**
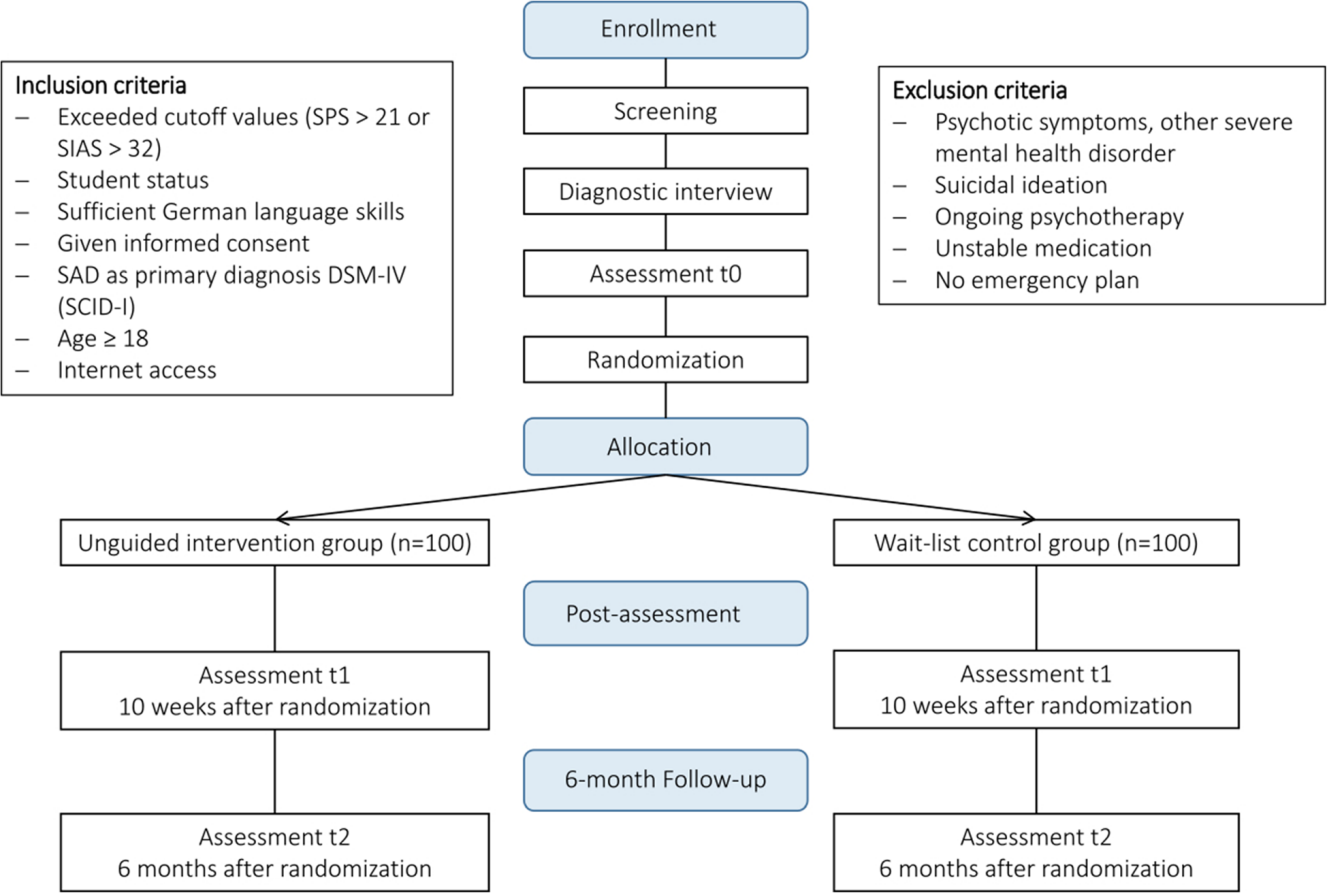
Study flow chart

We anticipate recruiting a total of *N* = 200 participants of which 100 participants will be assigned to each of the two conditions. Participants will be recruited in Germany, Austria and Switzerland. The recruitment strategy consists of various components: a study website, a promotional video, postings to Facebook and Internet forums and an email with information of the study sent to all German, Swiss and Austrian university psychological counseling centers and all students attending different universities based in Ulm, FAU, Bern, Dresden, Hagen, and Vienna. The study flow is illustrated in Fig. 1.

### Inclusion criteria

Participants will be included if they

- are a student,
- are at least 18 years old,
- have internet access,
- have sufficient German language skills as assessed via self-report (“Do you speak and understand German?”),
- exceed predefined cut-off scores in the SPS or SIAS,
- fulfill the diagnostic criteria of SAD according to DSM-IV assessed via a SCID-I diagnostic interview,
- have the ability to provide a written informed consent.

### Exclusion criteria

Participants will be excluded if they,

- show an acute suicidal risk according to the suicide item of the Beck Depression Inventory II (BDI II) (score > 1) or the diagnostic interview,
- have a history of psychotic or bipolar disorders,
- and are receiving psychotherapy at the time of entering the study.
- Prescription medications for anxiety and depression lead to an exclusion if the dosage was changed one month before the beginning of the study.

### Randomization

Two hundred participants will be randomly assigned. The allocation list is produced by a random number generator Randlist [9] which randomly allocates participants in a 1:1 ratio with a block size of 8 to either IMI or WLC. The list is operated by an independent researcher not otherwise involved in the study. This researcher has no information about the participants other than the participants’s trial ID numbers and will randomize the participants in the order of the incoming informed consent form. During the randomization process, the allocation will be concealed from participants and researchers involved in recruitment. Participants will not be blinded to study conditions.

### Internet-based self-help intervention with an additional session on fear of positive evaluation

The intervention is based on the well-established cognitive-behavioral treatment of Clark and Wells (1995) [51] and has been shown to be efficacious in several previous studies [27, 52–55]. It consists of nine text-based sessions, various exercises (e.g. attention training) and diaries (such as a diary to identify and question negative thoughts). Participants are advised to complete one session per week, review the exercises, and complete diaries. The approximate time required to complete one session is 60 min, and participants are encouraged to practice the strategies in their daily life. An overview of the sessions is summarized in Table 1. The original intervention was tailored to the university setting, for example, by providing case examples of socially anxious students.

**Table 1:** Session content for the SAD internet-based self-help program

Session	Intervention content
Session 1 Motivational enhancement	Reasons to initiate change, defining goals and recoding introspection of difficult social situations with help of an anxiety protocol
Session 2 Psychoeducation	Information on SAD and maintaining factors such as negative thoughts, safety behaviors and self-focused attention
	Development of an individual model for SAD
Session 3 Cognitive restructuring	Identification and modification of negative thoughts (dysfunctional assumptions) with the help of a thought diary
Session 4 Fear of positive (social) evaluation	Information about FPE (examples and explanatory models)
	Identifying FPE using a diary
	Recognizing the devaluation of own achievements, the benefit and the risks of positive evaluation using a diary
	Endorsing positive evaluations and emotions applying a self-compassion-interventions
Session 5 Self-focused attention	Various exercises to reduce self-focused attention
Session 6 Behavioral experiments	Planning and conducting in-vivo exposures
Session 7 Summary and revision	Summary of the key elements of the training
	Highlighting the importance of revising the exercises (e.g. in-vivo exposure)
Session 8 Healthy lifestyle and problem solving	Information about healthy lifestyle (e.g. sports, nutrition)
	Conveying problem solving skills
Session 9 Relapse prevention	Strategies to maintain the acquired skills
	Preparing for possible relapses

An additional module in session 4 not part of the original Clark and Wells program targets FPE. FPE is defined as discomfort and fear in reaction to positive feedback from others. The module contains psychoeducational material regarding the definition and etiology of FPE according to the bivalent fear of evaluation model [43] and the evolutionary model [44], as well as information on FPE-related cognitive strategies such as the disqualification of positive social outcomes (DPSO). A thought diary is introduced to identify and modify FPE-related cognitions, including perceived costs and advantages of positive evaluation. Additionally, the module contains exercises that aim at promoting self-compassion as well as the experience and acceptance of positive emotions which have been linked to a decrease of FPE [56].

Although this is a therapeutically unguided self-help intervention, guidance is provided via standardized automatic messages aiming to promote adherence. Adherence reminders follow procedures used in a number of previously conducted studies [57–59]. They consist of one positive reinforcement per session completion and one automatic reminder if participants do not log into the platform for more than one week. These automated reminders contain standardized personalized motivational messages, which strengthen participants’ adherence to the intervention.

### Procedure

Students who are interested in enrolling in the study will contact the study team through a contact form from a student mental health platform (i.e., on  www.studicare.com) or directly via email. If participants satisfy inclusion criteria they will be randomly assigned to either the IG or the WLC. Students who are assigned to the IG can initiate the online-based self-help intervention immediately after randomization. Students in the WLC will receive access to the program six months after randomization. The study includes three assessments: both groups are assessed at baseline (t0), immediately after completing the program (t1; 10 weeks) and at follow-up six months after randomization (t2). The assessment at t1 (10 weeks) is independent from treatment completion. Treatment adherence will be monitored after t1. Self-reported measures are collected using a secure web-based assessment system (UNIPARK, 256-bit encrypted [60]). This system allows for data validation (range checks, double data entries) to improve data quality. Additionally, participants’ SAD symptoms in IG will be assessed weekly via the intervention platform. The collected data will be stored securely.

### Measurements

A detailed overview of all measures at baseline (t0), 10-week post-treatment (t1) and 6-month follow-up is given in Table 2.

**Table 2:** Measurements and time of assessment

Instrument	Abbreviation	Aim	Time of assessment		
			t0	t1	t2
Clinician administered					
Structured Clinical Interview for DSM-IV Axis I Disorders	SCID-I	DSM-IV Axis I disorders	✔		✔
Self-report ratings					
Primary Outcome Measure					
Social Phobia Scale^a^	SPS	Symptoms of SAD	✔	✔	✔
Social Interaction Anxiety Scale^a^	SIAS	Symptoms of SAD	✔	✔	✔
Secondary Outcome Measure					
Liebowitz Social Anxiety Scale	LSAS-SR	Social anxiety symptoms	✔	✔	✔
Beck Depression Inventory II	BDI-II	Symptoms of depression	✔	✔	✔
Brief Symptom Inventory	BSI	Psychiatric symptoms	✔	✔	✔
Inventory of Interpersonal Problems	IIP-64	Interpersonal problems	✔	✔	✔
Fear of Positive Evaluation Scale	FPES	Fear of positive evaluation	✔	✔	✔
Disqualification of Positive Social Outcomes Scale	DPSOS	Fear of positive evaluation	✔	✔	✔
EuroQol (EQ-5D-5 L)	EQ-5D-5 L	Quality of life	✔	✔	✔
Assessment of Quality of Life (AQol)	AQoL-8D	Quality of life	✔	✔	✔
Client Satisfaction Questionnaire	CSQ-8	Client satisfaction			
Trimbos/iMTA Questionnaire for Costs associated with Psychiatric Illness	TiC-P	Cost-effectiveness	✔		✔
Credibility Expectancy Questionnaire	CEQ	Treatment expectancy			

t0 Baseline; t1 10 weeks; t2 6 months; Assessments ✔ = intervention and control group;  = intervention group ^a^ process measures assessed every 2 weeks

### Primary outcome measures

#### Symptoms of SAD

The primary outcome is SAD symptoms. SAD symptoms will be measured with two widely used measures, the Social Phobia Scale and the Social Interaction Anxiety Scale (SPS & SIAS; [61]). These two self-report questionnaires complement one another and are usually administered together. The SIAS assesses more general fears of social interaction (e.g., “I tense up if I meet an acquaintance in the street”), while the SPS focuses on fears of being judged by others during daily activities (e.g., “I become anxious if I have to write in front of others.”). Both scales consist of 20 items to be rated on a 5-point Likert scale (0= “not at all” to 4 = “extremely”). These two companion measures have been found to be valid, reliable and useful for clinical and research purposes [62]. Cronbach alphas for the SIAS and SPS range from 0.90 to 0.94 [63].

### Secondary outcome and process measures

#### Diagnostic status

The diagnostic status will be assessed with the Structured Clinical Interview for DSM-IV (SCID-I [64]). The interview will be conducted and recorded by trained raters (clinical psychologists or graduate students in psychology) via telephone at baseline and 6-months. The raters are blind to the condition the participants are assigned to. In order to ensure blinding, (a) participants receive information on the importance of not informing interviewers about the conditions they were assigned to, (b) raters receive a written reminder to not ask the participants for their randomization status, (c) written and verbal reminders for the participants before the interview; and (d) a documentation after the interview if the rater is still blind to treatment condition. The inter-rater reliability will be evaluated through a random selection of 10% of recorded cases.

#### Beck depression inventory II

Depression severity will be assessed using the Beck Depression Inventory II (BDI-II) [65]. The scale consists of 21 items each rated on a 4-point Likert-scale. Prior research has shown high reliability and validity in SAD clients [27].

#### Brief symptom inventory

General psychopathology will be assessed using the Brief Symptom inventory (BSI), which spans 9 dimensions, including insecurity in social situations, anxiety, depressiveness and compulsivity [66]. The BSI assesses symptoms within the past week and has shown robust psychometric properties [67]. The Global Severity Index (GSI), the overall mean score, will be reported.

#### Liebowitz social anxiety scale

The Liebowitz Social Anxiety Scale (LSAS) [68, 69] is a self-report scale that assesses fear and avoidance in 24 different situations. Thirteen of the situations relate to performance and the remaining items assess situations within the context of social interactions. Prior research has shown good to excellent reliability and validity (Cronbach’s alphas ranging from 0.83 to 0.94) [68].

#### Inventory of interpersonal problems

Difficulties in interpersonal behavior and sources of relational distress will be assessed using the Inventory of Interpersonal Problems (IIP-64) since they indicate assertiveness and passivity of participants. The instrument has eight dimensions and has shown adequate psychometric properties (Cronbach’s alphas ranging from 0.71 to 0.82) [70, 71].

#### Fear of positive evaluation

Fear of positive social feedback will be assessed using the Fear of Positive Evaluation Scale [72]. The FPES is a self-report measure consisting of 10 items and has shown good psychometric properties in clinical and healthy samples [48, 72].

The disqualification of positive social outcomes (DPSO) is a cognitive strategy which has been linked to FPE [43, 72]. This cognitive tendency is proposed to serve as a mental safety behavior in the context of FPE and will be measured using the Disqualification of Positive Social Outcomes Scale (DPSOS) [73]). The DPSOS is designed to measure the disqualification of positive outcomes on two dimensions, other-oriented attributions (e.g., “people will laugh at my jokes even if they are not funny”) and self-oriented attributions that refer directly to DPSO (e.g., “I frequently dismiss my own social successes and accomplishments”).

#### Quality of life

The Assessment of Quality of Life (AQol) [74] and the EuropeanQuality of Life 5 Dimensions 3 Level (EQ-5D-5L) instrument [75, 76] will assess quality of life. The AQol assesses eight dimensions (independent living, pain, senses, mental health, happiness, coping, relationships, self-worth) and allows for the calculation of separate sum scores for each dimension. The EQ-5D is a widely applied, valid and reliable measurement of quality of life. It consists of five items on a five-point Likert scale related to mobility, self-care, common activities, pain/discomfort and anxiety/depression. Additionally, this measure contains a visual analogue scale (VAS) to assess the respondent’s self-rated health status. Only the AQoL will be used as a secondary outcome, the EQ-5D will only be used for sensitivity analyses in the health economic outcome evaluation.

#### Cost measures

The Trimbos and iMTA Treatment Inventory of Costs in Patients with psychiatric disorders (TIC-P) [77] was adapted for the application to the German health care system and the specific target group. Direct medical costs (e.g., drugs), direct non-medical costs (e.g., transportation) and indirect costs (e.g., productivity losses) [78] will be assessed over a period of the previous 3 months. A catalog of German unit costs [79] will be used to calculate total health care costs on individual basis assuming that the majority of participants will be from Germany [80]. Indirect non-medical cost stemming from productivity losses due to presenteeism and absenteeism costs will be assessed with specific modules of the TiC-P. From a student’s perspective a monthly rate that students are paid due to the German Federal Law on Support in Education (BAföG) [81] is assumed covering the general cost for living and education in Germany. The intervention costs are estimated at €150 ($181) per participant. This tariff stems from a health care provider (GET.ON Institute) that offers comparable internet-based interventions. Including German VAT of 19%, interventions costs were €178.50.

#### Other measures

Other assessments will include demographic variables (e.g., age, gender, student status, etc.). Moreover, a version of the German Client Satisfaction Questionnaire (CSQ-8) [82] that was adapted to the online training context will assess the acceptance of internet-based interventions and global client satisfaction on the intervention [83]. Adherence to treatment will be evaluated by completion rate and time spent in intervention. The 6-item German version of the Credibility Expectancy Questionnaire (CEQ) [84] assesses the intervention’s credibility and outcome expectancies. Additionally, credit points based on the European Credit Transfer System (ECTS) [85] are assessed to evaluate the reduced academic productivity.

#### Process measures

Participants in the active conditions will be asked to rate their symptoms of SAD every two weeks (SPS & SIAS) in order to detect change in social phobic symptoms during the intervention.

### Power and sample size calculation

The study is powered to detect small to medium effect sizes of d = 0.4. The intended sample size of 200 participants will provide sufficient power to detect a significant standardized effect size (Cohen’s d) of 0.4 on the primary outcome variable (symptoms of SAD via SPS and SIAS) between the two conditions. The software Gpower [86] was used to calculate the sample size of *n* = 100 per group given a Bonferroni-adjusted (due to multiple testing) alpha error level of 0.025 for a one-sided test, a statistical power of 0.80 and an effects size of d = 0.4.

### Analysis

Analyses will be conducted and reported according to the Consolidated Standards of Reporting Trials (CONSORT) statement [87]. The results will be disseminated in peer-reviewed scientific journals. The depersonalized data will be analyzed based on the intention-to-treat principle. Missing data will be handled using multiple imputations with 10 estimations per missing value following recommendations of Little and Rubin [88] and Schafer [89]. Differences in continuous outcomes between the groups will be analyzed using analysis of covariance (ANCOVA), with pre-scores as a covariate and the post-scores as the dependent variable. Possible confounders (e.g. former use of psychotherapy) will be assessed and included as covariates if they should be associated with changes in the primary outcome. We will compute standardized effect sizes (Cohen’s d including the 95% confidence intervals for all effect sizes). We will also test differences in treatment response rates (50% relative symptom reduction), numbers of participants displaying reliable change (according to Jacobson and Truax [90]) as well as differences in symptom deterioration rates. Effect sizes between groups of dichotomous outcome variables will be expressed as number needed to treat and its associated 95% confidence intervals. All reported *p*-values are (one-sided) at a significance level of 0.025 for the primary outcomes and 0.05 for the secondary outcomes.

Moderators of the outcome will be analyzed on an exploratory basis using regression analyses, as well as region of significance procedures [91], in case of significant findings. Baseline variables considered for moderator analyses include: Sociodemographic and study-related characteristics (e.g. age, gender, nationality, full-time versus part-time students, study major, number of semesters on leave, number of semesters in total, ECTS points), baseline severity of social phobia (SIAS, SPS), depressive (BDI-II) and general psychopathological symptoms (BSI), interpersonal problems (IIP), fear of positive evaluations (FPE), health-related quality of life (AQoL), generalized vs. specific SAD, concurrent use of psychotropic drugs, prior mental health treatment, perceived treatment credibility (CEQ), comorbid depressive disorders (SCID), number of comorbid disorders (SCID), age of onset (SCID). Moderator analyses will not be adjusted for multiple testing, as the aim is to generate hypotheses to be tested in future confirmative studies. All directional hypotheses are tested one-sided, bidirectional hypotheses two sided.

### Economic evaluation

We will perform an economic evaluation from the societal and health provider perspectives that include all relevant costs and outcomes. A cost-effectiveness analysis as well as a cost-utility analysis will be conducted following guidelines from the International Society for Pharmacoeconomics and Outcomes Research (ISPOR) Good Research Practices Task Force Report and the recommendations of the Consolidated Health Economic Evaluation Reporting Standard (CHEERS) [92, 93]. In the cost-effectiveness analyses, symptom-free status via SPS and SIAS will be used as clinical outcome. For cost-utility analyses, quality-adjusted life years (QALYs) will be calculated based on the AQOL-8D. EQ-5D-5L will be used only in sensitivity analyses. We will compare both groups in terms of incremental costs and incremental effects, by calculating the incremental cost-effectiveness ratio (ICER). We will use bootstrapping (*N* = 5000) and 95% confidence intervals in percentiles to test the robustness of the ICER and to quantify the uncertainty surrounding the ratios. The results will be shown in a cost-effectiveness plane and in a cost-effective acceptability curve. Additionally, the robustness of the base-case findings will be tested with a multi-way sensitivity analysis (i.e. ± 50% intervention costs, costs outliers, EQ-5D-5L as alternative instrument to calculate QALYs).

## Discussion

SAD is a highly prevalent mental health disorder, also among university students. Affected students suffer from lower quality of life, high burden of psychological strain and reduced academic functioning. Internet-based interventions represent a low-threshold, easily accessible, and flexible treatment which may help to overcome the low utilization rates of those in need.

Within this study we intend to extend the evidence of internet-based self-help for SAD regarding efficacy and cost-effectiveness. Particularly, we want to strengthen the evidence of unguided treatments, since the question how much support is needed remains unanswered given prior mixed results. On the one hand, results from two meta-analyses [94, 95] found self-help programs with support to be more effective and with lower attrition rates compared to no support. Whereas a recent meta-analysis on internet-based guided cognitive behavioral interventions for anxiety disorders in general did not find such differences, indicating that effects between current guided and unguided treatments for anxiety might be smaller than previously anticipated [25].

There may be several other factors such as the intensity of screening procedures [52], the length, structure and comprehensiveness of the self-help program itself [96], the extent of support needed depending on the disorder [95] as well as human substitutes such as automated reminders [97], which systematically confound whether guided interventions lead to superior results compared to unguided interventions [98].

This study will have three noteworthy limitations. First, if this study demonstrates clinically relevant effects for unguided self-help for SAD, it has to be taken into account that this evidence will be based on an RCT, which is characterized by a highly structured participation and research attention. This is usually not the case when self-help interventions are offered in routine care. Since the securing of commitment represents an adherence-promoting element in self-help interventions, it has been argued that effect sizes of pure self-guided interventions found in RCTs are significantly overestimated for what can be expected in routine care [99], when no additional measures to increase adherence are applied. Hence, in order to achieve similar effects outside laboratory conditions, a clear concept for ensuring adherence through minimal guidance from a professional or lay health worker seems favorable.

Second, we will employ an open recruitment strategy in the general student population. Such a procedure mimics a public health approach of student mental health treatment barriers of face-to-face treatment pathways. However, results need to be interpreted cautiously in such a context and may not generalize to classical routine face-to-face clinical practice pathways. A previous study has found that patients undergoing internet-based treatment resembled national general samples more closely than samples from routine face-to-face mental health care [100].

Third, the cost assessment is based on a self-report instrument and it may be argued that self-report data are potentially less accurate compared to data collected directly from public registers. However, comparative studies of self-report questionnaires and diaries have found an acceptable comparability [101]. The remaining risk, however, is likely to be equal across treatments, making it unlikely that it will result in a bias between groups.

## Conclusion

To the best of our knowledge, this study will have the largest unguided RCT intervention group recruited for SAD treatment so far [15, 102]. This study will contribute to the evidence for the efficacy, cost-effectiveness and moderators of unguided internet-based self-help for social phobia in university students. If successful, this intervention would facilitate the adequate allocation of scarce resources and will provide valuable information of a public health approach of SAD treatment. When implemented on large scale, such interventions might help to reduce the immense burden associated with SAD in university students.

## Abbreviations

ANCOVA: Analysis of covariance
AQol: Assessment of Quality of Life
BAföG: German Federal Law on Support in Education
BDI-II: Beck Depression Inventory II
BSI: Brief Symptom inventory
CBT: Cognitive behavioral therapy
CEQ: Credibility expectancy questionnaire
CHEERS: Consolidated Health Economic Evaluation Reporting Standard
CSQ-8: German Client satisfaction questionnaire
DPSO: Disqualification of positive social outcomes
DRKS: German Clinical Trials Register
ECTS: European Credit Transfer System
EQ-5D-5L: European Quality of Life 5 Dimensions 3 Level instrument
FNE: Fear of negative
FPE: Fear of positive evaluation
ICER: Cost-effectiveness ratio
IG: Intervention group
IIP-64: Inventory of Interpersonal Problems
IMI: Internet- and mobile-based self-help intervention
ISPOR: International Society for Pharmacoeconomics and Outcomes Research
LSAS: Liebowitz Social Anxiety Scale
QALYs: Quality-adjusted life years
RCT: Randomized controlled trial
SAD: Social anxiety disorder
SCID: Structured Clinical Interview for DSM Disorders
SIAS: Social Interaction Anxiety Scale
SPS: Social Phobia Scale
TIC-P: The Trimbos and iMTA Treatment Inventory of Costs in Patients with psychiatric disorders (TIC-P)
VAS: Visual analogue scale
WHO: World Health Organization
WLC: Wait-list control group
WMH-ICS: WHO World Mental Health International College Student Initiative
